# Different Flavors of Astrocytes: Revising the Origins of Astrocyte Diversity and Epigenetic Signatures to Understand Heterogeneity after Injury

**DOI:** 10.3390/ijms22136867

**Published:** 2021-06-26

**Authors:** Alejandro Villarreal, Tanja Vogel

**Affiliations:** 1 Laboratorio de Neuropatología Molecular, Instituto de Biología Celular y Neurociencia “Prof. E. De Robertis” UBA-CONICET, Facultad de Medicina, Universidad de Buenos Aires, Buenos Aires 1121, Argentina; 2 Department of Molecular Embryology, Institute of Anatomy and Cell Biology, Medical Faculty, Albert-Ludwigs-University Freiburg, 79104 Freiburg, Germany; 3 Center for Basics in NeuroModulation (NeuroModul Basics), Medical Faculty, Albert-Ludwigs-University Freiburg, 79104 Freiburg, Germany; 4 Freiburg Institute for Advanced Studies (FRIAS), Albert-Ludwigs-University Freiburg, 79104 Freiburg, Germany

**Keywords:** reactive astrogliosis, astrocyte diversity, astrocyte heterogeneity, epigenetic mechanisms, astrocyte differentiation

## Abstract

Astrocytes are a specific type of neuroglial cells that confer metabolic and structural support to neurons. Astrocytes populate all regions of the nervous system and adopt a variety of phenotypes depending on their location and their respective functions, which are also pleiotropic in nature. For example, astrocytes adapt to pathological conditions with a specific cellular response known as reactive astrogliosis, which includes extensive phenotypic and transcriptional changes. Reactive astrocytes may lose some of their homeostatic functions and gain protective or detrimental properties with great impact on damage propagation. Different astrocyte subpopulations seemingly coexist in reactive astrogliosis, however, the source of such heterogeneity is not completely understood. Altered cellular signaling in pathological compared to healthy conditions might be one source fueling astrocyte heterogeneity. Moreover, diversity might also be encoded cell-autonomously, for example as a result of astrocyte subtype specification during development. We hypothesize and propose here that elucidating the epigenetic signature underlying the phenotype of each astrocyte subtype is of high relevance to understand another regulative layer of astrocyte heterogeneity, in general as well as after injury or as a result of other pathological conditions. High resolution methods should allow enlightening diverse cell states and subtypes of astrocyte, their adaptation to pathological conditions and ultimately allow controlling and manipulating astrocyte functions in disease states. Here, we review novel literature reporting on astrocyte diversity from a developmental perspective and we focus on epigenetic signatures that might account for cell type specification.

## 1. Introduction

The principal components of the human brain and spinal cord are neurons and glial cells that form and interact through complex networks. The complexity of these networks relies on the specialized subtypes of neurons and glia, which arise during development as a consequence of highly regulated processes of cellular differentiation and maturation. Among glial cells, astrocytes have become a major focus of interest in the field of central nervous system (CNS) pathology because astrocytes are one key component in the cellular responses to injury [1,2,3]. Astrocytes respond to injury through a process known as reactive astrogliosis [1,4]. Reactive astrogliosis involves gradual changes in astrocyte morphology, which can be observed for example by cellular hypertrophy. Alongside, the transcriptional programs of reactive astrocytes change. Ultimately, reactive astrogliosis can lead to glial scar formation around the injury site in the case of focalized lesions [5,6,7]. Reactive astrogliosis might have a proinflammatory component, in which astrocytes promote microglia activation and, in some cases, neurotoxicity [8,9,10]. Reactive astrogliosis is triggered in order to promote tissue regeneration. However, reactive astrocytes can also contribute to expansion of the damage, either actively or passively by stalling their homeostatic functions [1]. In either case, strategies aiming to reduce damage through the modulation of reactive astrogliosis should be directed to restore homeostatic astrocyte functions.

One of the main setbacks in understanding and controlling astrocyte response to injury lies in their cellular heterogeneity. Astrocyte heterogeneity after injury may arise, at least partially, in response to extracellular cues, which are specific to the nature and/or the location of the injury, and thus heterogeneity can be a consequence of exposure to different signaling molecules and/or gradients of the latter [1,5,11]. Basal astrocyte diversity, given by astrocyte subtype specification, might also contribute to this differential response. Astrocytes are in fact heterogeneous cells after development and, depending on the location and function, they diverge in their transcriptomes [12] or express different sets of proteins which might confer different response capacities [13,14,15].

In regard to development, astrocytes as well as neurons originate from neural progenitor cells (NPCs) and they develop unique and thus distinguishable features in response to extracellular cues. Such cues trigger intracellular signaling cascades which impinge on stable, epigenetic modifications on the chromatin. In general, epigenetic changes are crucial not only for cell lineage differentiation but also for cell type specification [16]. Interestingly, new evidence has also shown that astrocytes can present parental epigenetic imprinting that results in monoallelic parent-of-origin-specific gene expression [17]. Through the imprinting process astrocytes adapt different epigenetic backgrounds in specific alleles. However, a comprehensive view on the epigenetic signatures that underlie each astrocytic phenotype, is still pending. 

## 2. Astrocyte Diversity and Heterogeneity

### 2.1. Astrocyte Diversity in Healthy Conditions

Astrocytes are a very heterogeneous population of cells. A first description discriminated protoplasmic astrocytes in the gray matter from fibrillary astrocytes in the white matter. Later on in humans, at least nine types of distinct astrocyte morphologies have been described [18,19,20]. Astrocytes participate from and in synaptic functions, form and regulate the blood–brain barrier, regulate extracellular ions and clear the synaptic cleft from neurotransmitters, promote synapse formation and can even function as neural stem cells [21,22]. In each case, astrocytes adopt a specific phenotype. 

**Figure 1:**
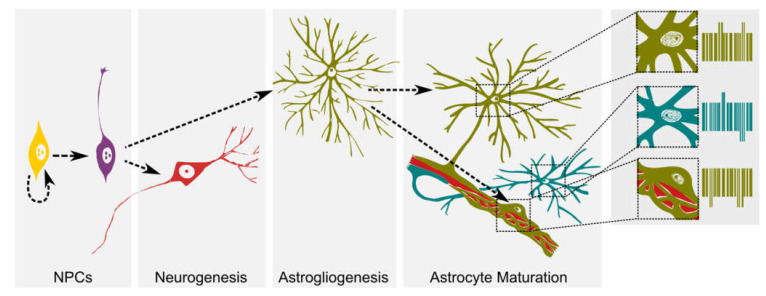
Graphical summary of neural differentiation and cell fate commitment starting from neural progenitor cells. In subsequent events of differentiation, proliferating NPCs will first differentiate into neurons (neurogenesis) and later on during development into astrocytes (astrogliogenesis). Astrocyte functional maturation occurs in a microenvironment dependent manner for example after contacting synapses or blood vessels. The figure shows two astrocytes of different clonal origin (green and blue) contacting a blood vessel (red) through end-feet processes and a third perivascular astrocyte. We propose that each of these astrocytes will have its own epigenetic signature (depicted here as a bar code), even when sharing the same clonal origin. Such an epigenetic signature may set the molecular bases of astrocyte diversity.

Interestingly, when human glial progenitor cells were engrafted into the mouse neonatal forebrain, spontaneous differentiation led to functional astrocytes, which enhanced synaptic plasticity and integrated into resident astrocyte networks through coupled gap junctions that propagated Ca^2+^ waves. Further, the engrafted astrocytes extended processes forming end-feet that reached blood vessels [23]. These observations further demonstrate that astrocyte acquire their final phenotype in a way that depends on location and function (Figure 1). Here, two main variables may be playing a role in sustaining the phenotype: microenvironmental cues and/or epigenetic mechanisms. 

The morphological distinction of astrocytes in combination with expression of specific marker genes allowed correlating structure and function [1,24,25]. However, recently the emergence of transcriptome analysis at single cell resolution provides an unbiased approach that contributes to and advances the understanding of astrocyte subtype diversity in terms of structure and function [12]. Batiuk et al. used a refined technique of astrocyte isolation from the adult mouse cortex and hippocampus followed by single cell RNA sequencing. This study reports on five distinct astrocyte subtypes. The mapping of each astrocyte cluster across brain regions using specific markers found for each cluster is a seminal part of this work. Interestingly, intra-regional differences were also reported, in which specific astrocyte subtypes were confined to specific cortical layers. Further, a specific astrocyte subtype was found in the subpial region of the cortex and in the dentate gyrus of the hippocampus. On the functional level, each of the described astrocyte subtypes possessed different Ca^2+^ transient properties, and one subtype had neurogenic properties. Another study analyzed 33 brain regions of 4 different primate species including humans. One finding of interest for this review is that human astrocytes are even more heterogeneous when compared to nonhuman primates [26]. This finding adds high resolution and detailed information to reported histological properties of human astrocytes [27].

Novel technological approaches allowed construction of a Large-area Spatial Transcriptomic (LaST) map, a working pipeline for the quantification of single cell gene expression in situ [13]. The authors found a layered distribution of astrocytes in the mouse cortex when addressing markers such as *Chrdl1* (Chordin like 1). Astrocytes expressing *Chrdl1* are located in neuronal layers 2–3 and 4; however, this pattern varies across regions suggesting astrocyte arealization across the cortex. Of particular note, astrocyte layers’ positioning occurs during brain development and lineage commitment, resembling the spatial distribution of neurons. As the spatial distribution of neurons is to some extent under the control of epigenetic mechanisms, it is tempting to speculate that these processes also impact astrocyte diversity and distribution [28,29,30].

### 2.2. Astrocyte Heterogeneity in Response to Injury

When experimentally addressing astrocyte response to injury through histological methods such as immunohistochemistry using astrocyte markers such as Glial Fibrillar Acidic Protein (GFAP), several features of the phenotype are shared between different insults, including hypertrophy and overlapping of astrocyte 3D domains [31]. Using a combination of GFAP with other markers such as aldehyde dehydrogenase-1 L1 (ALDH1L1), glutamine synthetase (GS), and aldolase-C (ALDOC) allows a broader overview of how different astrocyte subpopulations may respond to an insult. However, these markers are not enough to discriminate between astrocytes subpopulations in detail [1] and single cell analysis will contribute to better defining astrocyte subpopulations in reactive astrogliosis [12,14,32]. Astrocyte heterogeneity after injury has been widely analyzed and revised considering morphological and functional aspects [20,33,34,35,36], and here, we will focus in the following sections on the underlying molecular aspects.

When addressing astrocyte response to injury at a transcriptional or proteomic level, differences other than morphological variations are being uncovered. Zamanian et al. (2012) analyzed the transcriptome of astrocytes after brain ischemia or after systemic administration of the pro-inflammatory stimulus LPS (lipopolysacharide). Here, astrocytes were analyzed as one unique population of cells responding to injury. Both insults elicited the expression of pan-astrocyte markers; however, each injury promoted the expression of a specific set of genes. For example, LPS stimulation promoted the expression of genes involved in inflammation, and the authors suggested a detrimental role, while astrocytes in ischemia showed a protective profile of gene expression. Further works have linked the pro-inflammatory pathological phenotype to the expression and secretion of complement protein C3, which, once released, engages C3 receptors on neurons and microglia promoting toxicity and activation, respectively [8,9,37,38,39]. However, the concept of a detrimental vs. protective astrocyte is today under debate and it is likely that different astrocyte populations with different gene expression profiles coexist in the same microenvironment [1,32].

Anderson et al. (2016) analyzed the transcriptome of astrocytes that formed glial scars after spinal cord injury using a Ribo-tag technique for specific purification of mRNA undergoing translation in astrocytes. Here, the authors report that glial scar-forming astrocytes express multiple genes involved in supporting axonal growth [5]. 

These works revealed astrocyte heterogeneous response (molecular and functional) following different CNS insults (LPS, ischemia and spinal cord injury). However, since astrocytes were analyzed in bulk and not at the single cell level, it was not possible to distinguish between astrocyte subpopulations. In this regard, more recent findings using single cell transcriptomic analysis of astrocytes in experimental autoimmune encephalomyelitis (EAE) suggested that different astrocyte subtypes (anti-inflammatory astrocytes and pathological astrocytes) coexist in the same cellular response [32].

The authors of the studies above suggested that acquisition of different reactive phenotypes of astrocytes depended to a great extent on the extracellular milieu and microenvironment. However, as we propose in this article, intrinsic astroglial properties, which might be acquired during CNS development and confer cell-autonomous heterogeneity, might also have to be considered in any attempt to understand and describe heterogeneity in reactive astrogliosis.

## 3. Astrocyte Differentiation and Maturation

Astrocyte diversity that originates as a result of development is likely to be a source of heterogeneity in reactive astrogliosis when combined with a microenvironment caused by a specific insult or pathology. We consider that it further becomes relevant to understand how astrocyte diversity is achieved during differentiation and maturation.

Neural cells, namely neurons and glia, including both astrocytes and oligodendrocytes, arise from one multipotent progenitor population named neural progenitor cells (NPCs) [16,40,41,42]. NPCs are multipotent stem cells with the ability to proliferate, self-renew and differentiate into neurons, astrocytes and oligodendrocytes in a time-specific manner [41,43].

During CNS development, multipotent NPCs will first differentiate to neurons, directly or through intermediate neuronal progenitors [44]. After early neurogenesis, a switch in the neural tube progenitor domains occurs giving rise to glial cells in the brain and spinal cord [13]. 

As cells differentiate following intrinsic and extrinsic cues, their differentiating potential is gradually restricted becoming more committed to a specific cell population [16,45,46,47]. In this regard, differentiated astrocyte populations arrange into morphological domains across the CNS and it is likely that astrocyte diversity results from a combination of NPC heterogeneity and neuron-induced astrocyte patterning [13]. 

Cell type specification and fate commitment gradually occur in a time-dependent manner, giving rise to a stable conversion which generally is not spontaneously reversed, even after the external cues that induced differentiation are no longer available. Such cell identity-conferring stability can be mediated through specific epigenetic mechanisms [16,30,41]. 

We will briefly comment on the major ligands and signaling pathways that impinge on astrocyte differentiation and maturation, and highlight some of these pathways that involve downstream epigenetic mechanisms. 

### Signaling Pathways That Impinge on Astrocyte Differentiation and Maturation

The signaling pathway associated prominently with astrocyte differentiation is the JAK (Janus Kinase)-STAT (Signal Transducer and Activator of Transcription) pathway that is activated by cytokines, including LIF (Leukemia Inhibitor Factor), CNTF (Ciliary Neurotrophic Factor) and Cardiotrophin 1 [45] after engaging membrane receptors. In an *in vitro* model of human induced pluripotent stem cell differentiation, PDGF (Platelet-Derived Growth Factor) also activated JAK-STAT signaling, which was followed by astrocyte differentiation [48]. Activated STAT3 forms homodimers, which translocate to the nucleus and bind to consensus sequences at gene promoters suppressing neurogenesis and promoting astrogliogenesis [49]. Interestingly, STAT3 activation is furthermore involved in astrocyte phenotype conversion during reactive gliosis and glial scar formation [5,50,51]. 

Apart from JAK/STAT-signaling, the NOTCH-activated pathway controls astrogenesis. NOTCH-1 is a transmembrane receptor that binds to DELTA-LIKE and JAGGED ligands. Ligand binding promotes two consecutive cleavages of NOTCH, which releases its intracellular domain (NICD) into the cytoplasm. Cleaved NICD translocates to the nucleus where it binds to different cofactors, including transcription factors [52]. During embryonic development, NOTCH-signaling is involved firstly in the suppression of neuronal gene expression programs, promoting gliogenesis. Secondly, later on in development, it suppresses oligodendrocyte programs and concomitantly promotes astrogenesis [52,53]. These findings suggest that NOTCH signaling acts at different time points of astroglial differentiation. 

Bone Morphogenic Protein (BMP), a member of the TGF-β (Transforming Growth Factor-β) cytokine family that activates SMAD transcription factors, is a key factor of development [54], and it also impacts astrocyte differentiation [55,56]. TGF-β1 signaling has also been linked to modulation of neuronal and astroglial fate commitment in a spatial–temporal dependent manner [57,58,59].

Although the above mentioned signaling pathways affect astrogliogenesis individually, they can also act in concert. For example, a crosstalk between STAT3 and NOTCH pathways has been reported in which HES1 and 5, transcription factors upregulated by NOTCH signaling, interact with JAK2 and STAT3, and facilitate STAT3 phosphorylation and thus activation [60]. In this way, astrocyte differentiation by the NOTCH-HES pathway depends on STAT3. Similarly, BMP (BMP-2) and LIF pathways crosstalk through a complex formed by SMAD1 and STAT3, and which is bridged by acetyltransferase EP300. This complex results in synergistic signaling outcomes in the differentiation of astrocytes from NPCs [61,62].

Most of the currently published articles that address astroglial differentiation refer to astrocytes as a unique population of GFAP-expressing cells, and only few studies analyzed the origins of astrocyte heterogeneity. A work published by Bonaguidi et al. (2005) suggests that BMP and LIF signaling generate different kind of astrocytes with different features including different morphologies and cell division properties. Here, exposure to LIF promoted the differentiation of bi-tripolar astrocytes with proliferative capacities, while BMP promoted a stellated morphology and reduced cell divisions [63]. Recently, we have also addressed whether response to TGFβ-signaling might be a means to highlight astrocyte heterogeneity during brain development, which was indeed the case. Moreover, we determined that activity of FOXG1 (Forkhead box G1) discriminates astrocyte progenitors that generate different astrocyte lineages [59].

Although the activation of transcription factors is normally transient, their activity might have a long term impact on the regulation of gene expression programs. Therefore, understanding the downstream events controlling transcription factor networks beyond signaling pathways might be of importance. Therefore, in the next section we will comment on different epigenetic mechanisms that were linked to signaling pathways and transcriptional control involved in astroglial differentiation.

## 4. Epigenetic Signatures of Astroglial Differentiation

The term “epigenetics” is used to describe stable alterations in gene expression which may be triggered cell-autonomously or in response to environmental cues [64]. The sequential differentiation of NPCs along a specific lineage trajectory is epigenetically regulated [65,66,67].

Pioneering experiments by Frantz and McConnell (1996) in ferrets have shown the importance of cell-intrinsic mechanisms in neural cell fate restriction. Here, cortical progenitors at E42 (Embryonic day 42) that were developmentally primed to generate upper layer neurons were transplanted into an E32 cortex, in which progenitors generate mostly deep-layer neurons. The authors observed that the transplanted progenitors from the later embryonic stage were restricted towards generating upper layer neurons. Even when transplanted “late” progenitors were exposed to a change in the extracellular environment and were confronted with upper layer instead of deeper layer cues, they were incompetent to differentiate into deep layer neurons. At that time, intrinsic, yet undefined molecular mechanisms were proposed to be in place and responsible for restricting developmental potentials of progenitors with advanced age [68,69]. Heterologous grafting has not yet been conducted using astrocyte-committed NPCs, but such experiments would be desirable to enlighten developmentally determined variability in astrocyte fate and/or function. However, as already mentioned before, the engraftment of human glial progenitor cells into mouse brains led to astrocyte differentiation and emergence of different functions [23]. This suggests that in contrast to neurons, astrocytes seem to retain a higher degree of plasticity that allows acquiring the final phenotype in a context-dependent manner even after differentiation and maturation. Epigenetic timing of NPC differentiation and regulation of cell fate commitment are molecular mechanisms that govern a permissive genome stable enough to secure once made decisions, and sufficiently stable to allow adaptations to a changing environment.

**Figure 2:**
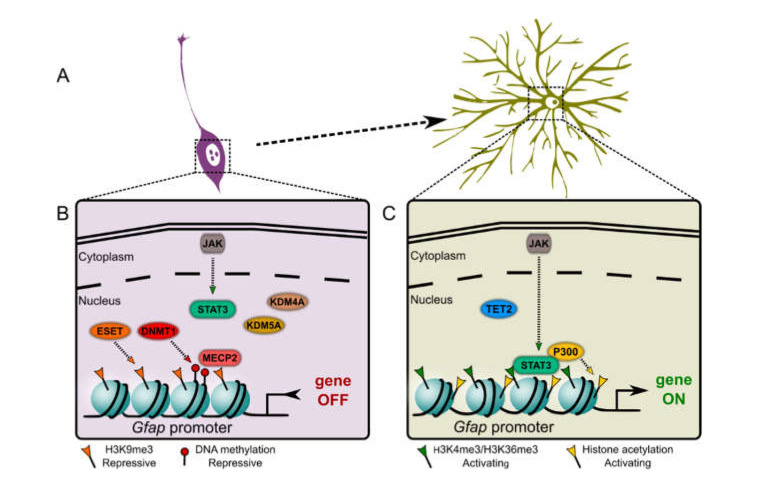
Graphical summary of the main epigenetic mechanisms described as regulators of astrocyte gene expression during astrocyte differentiation from NPCs. (**A**) In early astrocyte differentiation from NPCs, genes such as *Gfap* and *S100b* become activated while neuronal genes are repressed, giving rise to astrocytic phenotype. (**B**) At NPC stage DNMT1 methylates specific sites at astrocyte gene promoters (*Gfap* in the example) blocking STAT3 binding to the chromatin and inhibiting further transcription. Methylated DNA likely recruits chromatin reader MECP2 as described for *S100b* promoter. At this stage, histone demethylases KDM4A and KDM5A remove from the *Gfap* promoter the activating marks H3K36me3 and H3K4me3, respectively, contributing to gene repression. Further, histone methyltransferase ESET deposits the repressive mark H3K9m3 at the *Gfap* promoter. (**C**) Upon engaging specific extracellular stimuli TET2 promotes reduction of DNA methylation allowing STAT3 binding to *Gfap* promoter. STAT3 further recruits acetyltransferase EP300 promoting chromatin acetylation. In the absence of KDM4A and KDM5A, the levels of activating marks H3K36me3 and H3K4me3 increase at the *Gfap* promoter region. All these epigenetic mechanisms (and likely others) act in concert promoting *Gfap* expression during astrocyte fate commitment. ((**B**,**C**) are adaptations from [16]).

In the following we will address how DNA methylation and histone modifications, i.e., acetylation and methylations impact on NPC differentiation towards astrocytes and how they might contribute to astrocyte heterogeneity. The role of other epigenetic mechanisms, i.e., noncoding RNAs such as microRNAs, that play a pivotal role in astrocyte differentiation, was recently reviewed elsewhere [70]. A summary of the main epigenetic events described to regulate astrocyte differentiation is shown in Figure 2.

### 4.1. DNA Methylation

DNA methylation is the conversion of a cytosine to 5-methylcytosin (5mC) by covalent transfer of a methyl group to the C-5 position in the pyrimidine ring [71,72]. Methyl groups are added, also referred to as written, by DNA-methyltransferases (DNMTs), which use S-adenosyl-L-methionine (SAM) as methyl donor and are essential for mammalian development [71,72,73]. Three DNMTs are expressed in the mammalian brain, all of which are also essential for development in general: DNMT1 mainly participates in the maintenance of DNA methylation, whereas DNMT3A/B confer de novo DNA methylation [70,74]. Methylated DNA serves to recruit and dock of other proteins. For example, the transcriptional repressor MECP2 (methyl CpG binding protein 2) can bind to 5mC and thus “reads” this epigenetic mark [75,76]. Ten-eleven translocation (TET) methylcytosine dioxygenases are involved in DNA demethylation, as so called erasers. They catalyze the hydroxylation of 5mC to 5-hydroxymethylcytosine (5hmC), and the latter can either be erased during DNA replication or through oxidation and thymine DNA glycosylase (TDG)-mediated base excision repair after being reverted to cytosine [77].

In the context of astrocyte differentiation, it has been recognized that following STAT3 activation, this transcription factor bound the *Gfap* promoter and activated *Gfap* expression. However, accessibility of STAT3 and binding to the *Gfap* promoter were abolished if the respective genomic regions contained high levels of DNA methylation [78]. S100B is a calcium binding protein and its expression is widely used to describe astrocytes [1,79,80]. Unlike GFAP, S100B is also expressed by immature astrocytes [81]. The gene encoding for S100B is also subjected to regulation by DNA methylation, as it contains methylated sites at the promoter region that are demethylated when NPCs start to differentiate towards astrocytes. Following demethylation, the repressor MECP2 was released from *S100b* gene promoter and allowed activation of transcription [82].

*Dnmt1* is crucial for correct timing of astrogliogenesis. Its deletion in NPCs resulted in DNA hypomethylation and accelerated JAK-STAT pathway activation, which was accompanied by precocious astrogliogenesis of cortical NPCs [83]. Similarly, *Dnmt1* deletion in NPCs also promoted astrogliogenesis of hippocampal dentate gyrus granule progenitors [84].

Thus, generally, DNA methylation in NPCs is essential to preserve neuronal lineage differentiation by keeping astrocytic genes in a permissive silenced state. Further support for this view comes from a recent report in which the authors demonstrated using NPCs that DNA demethylation, conferred by TET2, of astrocyte lineage genes, including *Gfap,* favors differentiation into astrocytes [85]. Interestingly, the same work showed further that TET2-mediated de-repression of astrocyte-specific genes was counteracted by the bHLH (basic Helix-Loop-Helix) transcription factor OLIG2 (oligodendrocyte transcription factor) which promoted oligodendrocyte differentiation. OLIG2, by inhibiting *Tet2* expression, indirectly suppressed astroglial lineage differentiation.

### 4.2. Histone Modifications

Together with DNA methylation, different histone modifications have been reported to impact astrocyte differentiation. The transcriptional outcome of histone modifications varies and depends on the different types of post-translational modifications that include, for example, acetylation, methylation, phosphorylation, and ubiquitination, and locations of modifications within different histones and residues within the respective protein. We will focus mainly on histone acetylation and methylation, which are the most studied modifications as of yet that impact astrocyte differentiation.

Histone acetylation, independently to which lysine residue it is located within a histone protein, leads to an open state of the chromatin and thus actives transcription [86]. The enzymes responsible for writing acetyl groups are generically known as histone acetyltransferases (HATs), and their counterparts are the groups of histone deacetylases (HDACs) [86,87,88]. It is important to mention that histones are not the only targets of HATs and HDACs. Thus, in experiments using transgenic animals or inhibitors, the outcomes should be interpreted carefully and should take non-histone effects into account [89,90].

Of importance for astrocyte differentiation is the study of *S100b* gene expression as mentioned above. Here, the promoter of the *S100b* encoding gene was demethylated upon astrocyte differentiation and it gained simultaneously acetylated H3 [82]. Similarly, the promoter of the *Gfap* encoding gene gained also acetylated H3 after exposing NPCs to retinoic acid, which promoted astrocyte differentiation [91]. Retinoic acid, after binding to the retinoic acid nuclear receptors, recruited HATs to the retinoic acid response elements at the *Gfap* promoter and the presence of HATs changed the chromatin configuration to the open state that allowed STAT3 binding and transcriptional activation.

STAT3 interacts directly with HATs as it is recruited by cAMP response element-binding protein (CREB) binding protein (CBP) and the transcriptional coactivator EP300. CBP/EP300 have acetyltransferase (HAT) activity [92,93] and the complex acetylated H3K9 and H3K14 at the *Gfap* promoter. These modifications facilitated transcriptional activity, which was shown using the NTera-2 cell line that differentiated into an astrocyte-like lineage [94].

The ablation of HDAC3 in glial progenitor cells promoted astrogenic differentiation with concomitant loss of oligodendrocytes [95]. This finding indicates that specific patterns of acetylation are required for controlling and balancing the astrocyte/oligodendrocyte fate switch. Of note, pharmacological inhibition of HDACs modulated splicing of the *Gfap* gene product in human astrocytes, showing that even after differentiation, HDAC activity regulates *Gfap* expression [96]. It is, however, unclear as of yet whether the underlying molecular mechanism involves acetylated histones or whether the HDACs target other proteins, i.e., components of the splicing machinery.

TGF-β signaling has been linked to astrocyte differentiation through the activation of SMAD proteins [57,59,97]. However, a broader view beyond the core SMAD proteins might be possible since it has been recognized that they interact with chromatin remodelers such as EP300/CBP acetyltransferase and HDACs. Thus, further mechanisms of controlling transcription are worthy to be explored during acquisition of astrocyte identity [98].

Regarding histone methylations and transcriptional outcome the picture is much more complicated, as several marks are associated with active transcription such as methylations of histone 3 at lysines 4 or 36 (H3K4me or H3K36me, respectively), while methylations at other residues in H3 confer gene silencing, for example H3K27me and H3K9me. Further, lysines can be subjected to mono-, di- or trimethylation (me1, me2 or me3, respectively) with different outcomes in regard to transcription [99].

NTera-2 cells that differentiated into an astrocyte-like lineage, had not only higher levels of acetylated H3 through STAT3-recruited HATs but also increased levels of the activating mark H3K4me3 at the *Gfap* promoter [94].

Polycomb group (PcG) proteins modulate the chromatin structure and generally repress transcription [100]. PcG proteins are part of the Polycomb Repressive Complex 1 and 2 (PRC1 and PCR2). PRC2 contains enhancer of Zeste homolog 2 (EZH2), a methyltransferase that methylates histone 3 at lysine 27 (H3K27me). The H327me3 mark can serve as a platform for PRC1 recruitment. PRC1 complexes contain the Ring1 ubiquitin ligases which are required for persistent PRC-mediated repression [101,102,103]. It was reported that PcG-mediated repression of Neurogenin 1 (*Ngn1*) occurs in the late stages of cortical development, when progenitor activate their astroglial potential, whereby neurogenesis is repressed and astrogenesis promoted [16,104].

However, the actions of PcG-mediated control of lineage determination of NPCs might be highly context-dependent, especially in a temporal dimension, as the conditional depletion of *Ezh2* in cortical progenitors in early development during their neurogenic phase using an Emx1-Cre mouse driver line, accelerated gliogenesis and glial differentiation towards GFAP-positive astrocytes [105].

H3K9me is a repressive mark known to promote heterochromatinization by recruitment of heterochromatin protein 1 (HP1) and it forms one barrier for cellular reprogramming [106,107]. Histone methyltransferase ESET (SETDB1 or KMT1E) confers the repressive mark H3K9me3. The ablation of ESET reduced H3K9me3 at *Gfap* promoter, and reduced neurogenesis by enhancing astrocyte formation [108], and thus ESET might be involved in regulating the neurogenic/gliogenic switch during development.

Histone demethylases KDM4A (JMJD2A) and KDM4C (JMJD2C) removed H3K9me from the promoter of the neurotrophic factor *Bdnf* (Brain Derived Neurotrophic Factor) gene at the same time that they removed the activating mark H3K36me3 from the *Gfap* gene body in NPCs. Together, this epigenetic remodeling promoted neuronal differentiation. Further, KDM4A/C depletion facilitated astrocyte differentiation at the expense of neuronal differentiation [109]. Here, one enzyme was responsible for removing two different and opposing histone marks at two different genomic regions. An increase in GFAP-positive astrocytes was also observed in KDM4C hypomorphic mutant mice [110].

Some of these findings might seem contradictory since the ablation of methyltransferase ESET and demethylase KDM4A for H3K9me led both to an increase in astrocyte differentiation. However, these studies did not address global changes in astrocyte transcriptome after enzyme ablation. The expression of other genes involved in the neurogenic/gliogenic switch might be affected by these genetic manipulations and are being neglected. To analyze a broader gene network, new studies will benefit from mRNA sequencing and concomitant profiling of the epigenetic landscape.

Histone demethylase KDM5A removes the activating mark H3K4me3 from the *Gfap* promoter. KDM5A knockdown in NPCs promoted H3K4me3 at the *Gfap* promoter and concomitant astroglial differentiation, which suggested that KDM5A demethylase activity repressed astrogenesis [111].

Histone ubiquitination also plays a role in astrocyte differentiation. Ring finger protein 20 (RNF20), also known as an E3 ligase, catalyzes the monoubiquitination of histone 2B at lysin 120 (H2BK120ub). RNF20 in cooperation with MOF (Males absent On the First) acetyltransferase, which acetylates H4K16, promoted transcriptional activity of STAT3 favoring astrocyte generation [112].

In some cases, the deposition of a histone modification might alter the deposition of a second one, a phenomenon known as histone-to-histone crosstalk [113]. Interestingly, H2BK120ub is involved in such histone–histone crosstalk events in cellular differentiation processes [114,115,116] and astrocyte differentiation might also involve such mechanisms.

A recent work conducted by Tiwari et al. (2018) used a combinatorial approach to analyze transcriptional regulation of differentiating astrocytes at a genome wide level. The authors implemented mRNA-seq and ChIP-seq at different differentiation stages of the astroglial lineage. This allowed the study of different epigenetic marks such as H3K27ac and H3K4me1, found at active or activatable enhancers, and the correlation of the marks with the expression of downstream genes [117]. In this way, the authors not only described the dynamics of the marks included in their study, but they also identified different transcription factors which are stage- and astrocyte-specific. Thereby the authors uncovered new regulatory networks of astrocyte differentiation that can be exploited in more detail to entail the basis of astrocyte development, which lacks the necessary detailed insight as of yet to fully understand their role both in pathology and normal function.

When all of the described aspects are taken together, it becomes clear that the switch from neurogenesis to astrogenesis depends on epigenetic mechanisms occurring at the chromatin level, that regulate gene expression programs involved in astrocyte fate commitment. However, much less is known when it comes to our current understanding, which mechanisms regulate lineage subtype specification in relation to astroglial function. It might be possible that astrocyte subpopulations are under the control of a combination of different epigenetic mechanisms triggered by specific location/environment and activity during maturation (Figure 1).

### 4.3. Contribution of Epigenetic Parental Imprinting to Astrocytes Diversity

A very interesting and recently discovered source of astrocyte diversity has its basis in parental inheritance. In a way which is not fully known, specific alleles in astrocytes are decorated with epigenetic marks conferring a parental-specific gene expression. This imprinted expression pattern may lead to a diversity of astrocytes across brain regions with genes carrying either maternal or paternal imprinting and differential gene activity [17]. Some of the described imprinted genes affect cell survival. The term “astrocyte resilience” was recently defined by Escartin et al., 2021 as the set of successful astroprotective responses that maintain cell-intrinsic homeostatic functions in neural circuits while promoting both neuronal and astrocyte survival. In light of this novel finding, epigenetic parental imprinting of survival genes in astrocytes may contribute to the resilience of specific subpopulations.

## 5. Experimental Approaches to Determine Astrocyte Epigenetic Signatures

Previous works have addressed the role of specific epigenetic mechanisms mainly during the neurogenesis/gliogenesis switch by deleting or inhibiting enzymes and addressing the relevance of certain epigenetic marks at the promoter of genes involved in astrocyte differentiation. Although new studies were able to analyze astrocyte subpopulations by addressing transcriptomic programs [12,14], to date, no work has described astrocyte subpopulations and diversity in terms of epigenetic signatures. We will comment in this section on certain techniques that might contribute in the understanding of astrocyte diversity in terms of epigenetic signatures, although some of them still represent a major challenge.

### 5.1. Function-Associated Signatures

Single cell RNA-seq and analysis allows clustering of isolated astrocytes and potentially correlating each cluster to a specific function, based on the transcriptional signature. Further, it is possible to analyze after single cell ATAC (Assay for Transposase-Accessible Chromatin)-seq the chromatin landscape in diverse astrocyte clusters to determine open and closed regions, especially enhancers that are used to establish or maintain cellular and functional heterogeneity [118,119]. This approach has already been employed and has led to the proposal of novel gene networks that regulate the emergence of different astrocyte subpopulations that coexist in reactive astrogliosis [32].

Using single cell analysis followed by ChIP-seq with antibodies against different epigenetic marks, could allow the assignment of a histone code to the astrocyte clusters mentioned above. Single cell ChIP-seq has been used either when starting from cultured cells [120] or from more complex tissue such as breast cancer patient-derived xenographs [121]. It was very recently shown that these kind of approaches can be adapted to specifically analyze brain-derived cells [122]. However, single cell ChIP-seq still represents major challenges compared to single cell mRNA- and ATAC-seq when analyzing individual cells. However, we also want to point to the relative novel techniques of CUT&RUN and CUT&TAG [123,124,125] that allow downscaling towards a degree that might be necessary to follow molecular alterations as hallmark of astrocyte heterogeneity.

### 5.2. Location Associated Signatures

In the above mentioned approaches astrocytes are isolated from their microenvironments. Although the analysis of gene expression may be highly indicative of the function of each cluster, astrocyte origin is lost. It is possible then to obtain samples (astrocytes) directly from the tissue by conducting spatial transcriptomics that allow, through a combination of different approaches, regional positioning of cell clusters obtained from the single cell RNA-seq [126]. Fan et al. (2018) isolated single brain cells from 22 brain regions and, together with the analysis of region-specific gene expression, managed to position in the developing brain the obtained cell clusters [127]. These kind of approaches can also be conducted using laser microdissection as shown by Baccin et al. (2020) [128]. In this work, the authors addressed the spatial organization of bone marrow niches at a molecular and cellular level. Spatial transcriptomics could then extend, as mentioned above, towards techniques to address epigenetic signatures. We mentioned above that ChIP-seq is still challenging to conduct at a single cell level. However, it has been shown that spatio–temporal differences of epigenetic signatures might be controlled by using a CRISPR (clustered regularly interspaced short palindromic repeats)-dCAS (dead CAS) based epigenome editing approach [129]. Here, a dCAS without catalytic activity is fused to the catalytic domain of chromatin remodelers, which can be guided to specific regions in the genome. Such a technique is still in early development for its application in the CNS and tissues in general; however, expressing dCAS under different astrocytic gene promoters and delivering it by *in utero* electroporation could represent a promising approach for conducting gain/loss of specific epigenetic marks in astrocytes.

### 5.3. Human Studies

Analysis of astrocyte epigenetic diversity can also be carried out from human biopsies or necropsies. However, in such cases the homeostatic astrocyte phenotype has to be addressed since aging and clinical conditions of the human donors might change the epigenetic signature of astrocytes. A promising approach involves the use of human induced pluripotent stem cells and generation of organoids. However, in such case it is still difficult to recreate the conditions and architecture of a healthy human brain, as the epigenome might be altered as well during extended cultivation periods and differentiation paradigms [26,130]. Further, there is still controversy on to what extent organoid systems recapitulate human brain development in terms of cell types. In this regard, a recent work using single cell RNA-seq from developing human brain suggested that neural progenitor heterogeneity is higher than previously described [131]. Interestingly, the authors further compared the obtained data sets with single cell RNA-seq data obtained from human organoids and observed that only at later stages of growth in culture, organoid cells better resemble their corresponding cortical progenitor counterparts in the brain. These results suggest that to some extent, organoids might be usable to study cell heterogeneity. Even if the use of organoids may limit the study to a specific time frame of development, it still provides an experimental platform for conducting loss/gain-of-function experiments to interfere with specific epigenetic mechanisms and to address astrocyte heterogeneity during differentiation.

## 6. Final Remarks

Astrocyte basal diversity contributes to the heterogeneity of reactive astrogliosis, which is still a major focus of interest in the field of neurodegeneration. Different populations of astrocytes display different transcriptional programs, which are probably epigenetically regulated and, although several epigenetic mechanisms have been described as intrinsic regulators of astrocyte differentiation, the epigenetic signatures of astrocyte subtype specification still remain unknown.

Basal epigenetic diversity can be acquired during embryonic development when astrocytes differentiate and functionally maturate in a “healthy” microenvironment. Knowing the epigenetic signature of each astrocyte subtype might be relevant to fully restore homeostatic capacities of surviving astrocytes in an injured brain or spinal cord.
